# Optimization of Multi-Objective Mobile Emergency Material Allocation for Sudden Disasters

**DOI:** 10.3389/fpubh.2022.927241

**Published:** 2022-07-14

**Authors:** Jianxun Li, Haoxin Fu, Kin Keung Lai, Bhagwat Ram

**Affiliations:** ^1^School of Economics and Management, Xi'an University of Technology, Xi'an, China; ^2^International Business School, Shaanxi Normal University, Xi'an, China; ^3^Centre for Digital Transformation, Indian Institute of Management Ahmedabad, Ahmedabad, India

**Keywords:** sudden disasters, mobile emergency facility, supplies to allocate, multi-objective programming, emergency management

## Abstract

The mobile emergency system is a new emergency mode that provides a solution to deal with increasingly frequent sudden disasters by reasonably allocating mobile emergency facilities and optimizing the allocation of mobile emergency materials. We consider mobile emergency cost and mobile emergency time as two objective functions. This paper establishes a multi-objective mobile emergency material allocation model, and transforms the multi-objective. We choose the emergency material transportation path for coding, and apply the hybrid leapfrog algorithm for material allocation to obtain the optimal solution. Finally, the feasibility of the model is verified by taking Zhengzhou urban area under the “21.7” severe rainstorm and flood disaster in Henan Province. The result analyses show that the model can correspond to each stage of mobile emergency material allocation based on the value of cost preference, and put forward suggestions on the location of mobile emergency facilities and the amount of material allocation.

## Introduction

In recent years, several sudden disasters have disrupted the face of human society. These cause economic losses, unstable industries, and other aspects of livelihoods and safety. The present technology is inefficient to predict the outbreak time, the severity of sudden disasters, the post-disaster emergency response time, and the cost factors accurately. Thus, a mobile emergency application has become the first choice to solve these problems. The mobile emergency supplies a dynamic, convenient, and rapid emergency response system for sudden disasters. Based on the mobile emergency facilities that are not limited by geographical location, a mobile emergency can provide emergency command, event disposal, material transportation and other services, which can achieve more timely, agile and accurate material deployment services. Mobile emergency facilities are equipped with medical, communication, rescue, detection, engineering and other equipment, and usually take various types of emergency vehicles as the carrier, with the advantages of variable location, flexibility, and pre-decision. In contrast to the traditional emergency response mode, the mobile emergency facilities can effectively control the transport time, and cost of emergency supplies, and provide services in the disaster front and remote areas, respectively, to save lives and property to the greatest extent. To sum up, the mobile emergency response is a new type of emergency response mode that responds quickly to emergencies through the pre-stored mobile emergency facilities. Therefore, the deployment of emergency supplies by mobile emergency facilities is an essential link of mobile emergency response.

The existing studies on the allocation of emergency supplies can mainly be divided into two categories: Location of emergency facilities and Distribution of emergency supplies. The relevant studies can be traced back to the p-center. The p-center issues were proposed by Hakimi (1) and the collective coverage issues were proposed by Roth (2). The most subsequent studies on the allocation of emergency supplies are based on these approaches. To carry out in-depth research with different objectives and different constraints as the center, typically, Sheu (3) utilized the fuzzy clustering method to classify disaster-hit points to ensure that the emergency supplies are firstly delivered to disaster-hit points with higher priority. Considering the occurrence probability, demand for emergency supplies and damage degree of different disasters, Zhang (4) proposed the optimal emergency reserve allocation strategy for government agencies by adopting scenario-based integer programming method. Based on the external conditions of multi-objective, multi-path, and multi-materials, Najafi et al. (5) suggested the post-disaster transportation in large scale and emergency rescue operations through a time optimization algorithm. Based on fairness, timeliness and economy, Bai (6) designed a multi-objective model for facility location, vehicle routing and material allocation decision problems. He et al. (7) focused on 3D printing emergency supplies distribution to improve the shortage of post-disaster medical supplies, and to explore its supply-demand relationship and efficient distribution path. Pradhananga et al. (8) demonstrated the multi-source emergency supplies that are an effective approach to reduce the social costs. On the premise of using five random scenarios to represent differentiated post-disaster situations, Cavdur et al. (9) proposed a two-stage stochastic programming method to solve the problem of minimizing the total distribution distance, unmet demand and the total number of facilities. Zheng et al. (10) realized the importance of pre-deployment of emergency supplies to improve the response speed and reduce the disaster impact and different heuristic algorithms were used to obtain optimal solutions of multi-objective optimization problems. In terms of the design, construction and application of mobile emergency facilities, Li et al. (11) established a two-stage planning model with the minimal total cost keeping as the objective function to seek a reasonable scale and location of mobile emergency facilities and solved the model using the ant colony calculation methods. Later, Li et al. (12) solved the path planning of mobile emergency facilities under the maximization of total service demand with the help of robust optimization and genetic algorithm based on the uninterrupted characteristics of emergency services. Friggstad and Salavatipour (13) studied the service processes of mobile facilities to minimize the cost of mobile services. Focusing on psychological changes of victims, Geng et al. (14) incorporated the cost of victims' pain perception into the total cost of emergency relief and built a government-enterprise joint positioning and allocation model for emergency supplies. Chen et al. (15) determined the best service location of medical facilities by integer programming and network differentiation mode, taking into account the infrastructure transportation facilities. In addition, Geroliminis et al. (16) proposed the optimal deployment model and heuristic solution algorithm of mobile emergency response units based on urban traffic networks.

The allocation of emergency supplies has been widely discussed, and several models have been developed to maximize the service coverage rate, minimize the shortest emergency time, etc. Such models effectively solve the site selection problems, transport problems and other issues related to the emergency response processes. With the gradual development of mobile emergency services, mobile emergency centers cover all kinds of cities. The mobile emergency center gives fast mobile and highly efficient emergency supplies mixing ability. However, the related research is still in the preliminary exploration stage at present due to the lack of mobile emergency response time, response efficiency, and response cost for an overall consideration of material blending optimization schemes. For these reasons, the present article relies on a mobile emergency facilities mode that is flexible, and independent from others. The cost important degree is introduced to distinguish between different stages of emergency decision-making requirements. The sudden disasters mobile emergency supplies allocation optimization model is presented through the multi-objective objective optimization problem. This problem is transformed into a single objective optimization problem to find the optimal solution. The hybrid leapfrog algorithm is utilized to solve the single-objective problem. The proposed model is verified in a rare sudden heavy rainfall disaster scenario in Henan Province.

## Multi-Objective Mobile Emergency Material Allocation Model

### Problem Description

After the sudden disasters, there is a need to move quickly to the disaster areas and their surrounding areas to carry out the emergency rescues. However, due to a large number of emergency supplies demand points, wide distribution, rapid demand change, and complex surrounding geographical conditions, the deployment mode from the headquarters of mobile emergency supplies storage to the demand points will face unimaginable difficulties in data processing and decision-making complexity. Therefore, the easily accessible and spacious emergency supplies service points are considered transfer points and buffer zones for mobile emergency supplies deployment. The emergency vehicles are responsible to deliver supplies to the emergency service point. They also deliver the supplies to meet the demand of neighboring points. The core question is how headquarters perform these activities in the given stipulated time, space, and resource constraints. The docking location of mobile emergency facilities, the allocation route, and the number of response materials are determined to consider the uncertainty of material demand at demand points and minimize the deployment time and cost. It is also necessary to establish a mobile emergency response system that includes the mobile emergency supplies storage center, mobile emergency facilities, mobile emergency supplies service points and mobile emergency supplies demand points to control all kinds of sudden disaster scenarios. In this system, emergency supplies begin from the emergency center and enter the disaster area through the mobile facilities. After arriving at the predetermined service point, they will radiate to certain surrounding areas with themselves as the center to ensure a sufficient supply of material demand points in the region. Each service point follows the principle of distance minimization to serve each demand point. In addition, when the service capacity of mobile emergency facilities in a service point is saturated, or the response speed is slow and the response cost is high, idle mobile emergency facilities can be introduced to provide services for the demand points of emergency supplies. Thus, the flexibility of material supply is improved through the service points of emergency supplies, to maximize the saving of lives and property. Considering the complexity and uncertainty of emergencies, the following assumptions are proposed to improve the realistic fitting degree of the model:

**Assumption 1** The mobile emergency response system should meet the needs of all emergency supplies demand points.

**Assumption 2** All kinds of mobile emergency supplies should be transported in standardized containers one by one (not mixed).

**Assumption 3** The mobile emergency facilities should be able to reach planning service points independently, and mobile emergency facilities should have no quantity and capacity limitations.

### Model Notation and Formulations

The deployment plan of mobile emergency supplies is directly related to the disaster scenarios. The differences in each type of sudden disaster scenario affect the emphasis of mobile emergency deployment decisions. However, no matter what kind of disaster scenario appears, the deployment of mobile emergency supplies needs to follow uniform rules and standards. The mobile emergency supplies allocation model can be considered a multi-objective planning problem. This model reasonably allocates multiple supplies and mobile facilities among storage center collection, mobile emergency facilities service points, and emergency supplies demand points collection. The symbols needed are defined as follows:

**Notations**

*O*: collection of mobile emergency storage centers indexed by o,
*I*: collection of mobile emergency facility service points indexed by *i*,
*J*: collection of emergency supplies demand points indexed by *j*,
*K*: types of mobile emergency supplies indexed by *k*,
*f_i_*: fixed cost of opening mobile emergency facility service points,
$c_{oi}^{k}$: unit cost of transportation of mobile emergency supplies *k*from mobile emergency supplies storage center o toemergency facility service point *i*,
$c_{ij}^{k}$: unit transportation cost of mobile emergency supplies *k* from emergency facility service point i to emergency supplies demand point *j*,
$t_{oi}^{k}$: single transport time of mobile emergency supplies *k* from mobile emergency supplies storage center o to emergency facility service point *i*,
$t_{ij}^{k}$: single transport time of mobile emergency supplies *k* from emergency facility service point *i* to emergency supplies demand point *j*,
*t_i_*: mobile emergency service point *i* times to prepare for emergency activities,
$s_{i}^{k}$: unit storage cost of category *k* emergency supplies at mobile emergency service point *i*,
$h_{maxk}^{k}$: the maximum quantity of category *k* emergency supplies that can be provided by the mobile emergency storage center o,
$d_{j}^{k}$: the quantity of demand for category *k* emergency supplies at demand point *j*,
${ϴ}_{j}^{k}$: the quantity of unmet for category *k* emergency supplies at demand point *j*,
$\lambda_{j}^{k}$: penalty coefficient of unmet unit of demand point *j* for category *k* emergency supplies.

**These are the following decision variables:**

*x_i_: x_i_ = 1represents the mobile emergency facility* service point, otherwise *x_i_ = 0*,
$y_{j}^{k}$: quantity of category *k* emergency supplies stored at mobile emergency facility service point *i*,
$z_{oi}^{k}$: $z_{oi}^{k}$ = *1 represents the transport of category *k* emergency* supplies from storage center o to emergency facility service point *i*, otherwise $z_{oi}^{k}$ = *O*,
$z_{ij}^{k}$: $z_{ij}^{k}$ = *1 represents the transport of category *k* emergency* supplies from emergency facility service point *i* to, emergency supplies demand points *j*, otherwise $z_{ij}^{k}$ = *O*,
$n_{oi}^{k}$: the amount of category *k* emergency supplies transported from the mobile emergency storage center o to the emergency facility service point *i*,
$n_{ij}^{k}$: the number of category *k* emergency supplies transported from emergency facility service point *i* to emergency supplies demand point *j*.

The research content of the multi-objective emergency material allocation model for sudden disasters covers the whole process of emergency material allocation after the sudden disasters. The fundamental goal is to control the cost of emergency material allocation as much as possible under the premise of minimizing the time of emergency material allocation and ensuring the safety of the lives and properties of the affected people. Due to the unpredictability and variability of sudden disasters, the proposed model solves the optimal service site selection scheme of mobile emergency facilities and the optimal quantity of materials allocation under the external conditions of mobile emergency facilities based on the available uncertainties. This model focuses on the simple land transport distribution mode of multiple types of post-disaster supplies, without considering the connection time and deployment loss of mobile emergency equipment in multi-modal transport, and the distribution and distribution process after the deployment of supplies is not included in this study. We now start to formulate the model. The emergency facilities should be the total costs *C* and the contingency total time is *T* in the same height. The total cost *C* includes the fixed cost *C*_1_, storage cost *C*_2_, transportation cost *C*_3_ and penalty costs *C*_4_. The fixed cost $C_{1}=\underset{i}{\sum }x_{i}f_{i}\;$ refers to various fixed start-up cost required by mobile emergency facilities to carry out emergency work after supplies reach the service point of emergency facilities. The storage cost $C_{2}=\underset{ik}{\sum }y_{i}^{k}s_{i}^{k}\;$ is used to represent the cost of inventory and supporting support for material transfer during a mobile emergency. The transportation cost $C_{3}=\underset{oijk}{\sum }\left(z_{oi}^{k}n_{oi}^{k}c_{oi}^{k}+z_{ij}^{k}n_{ij}^{k}c_{ij}^{k}\right)\;$ includes the total cost of emergency supplies going through two stages: from the storage center to the service point and from the service point to the demand point. The penalty cost $C_{4}=\underset{ik}{\sum }\theta_{i}^{k}\lambda_{i}^{k}\;$ is the loss that continues to be difficult to assess directly after emergency supplies fail to reach the point of need. The total emergency time *T* should be composed of turnover time *T*_1_, transport time *T*_2_ and penalty time *T*_3_. The turnaround time $T_{1}=\underset{i}{\sum }t_{i}$ refers to the time required for emergency supplies to enter the service point of the mobile emergency facility and make it serviceable. The transport time $T_{2}=\underset{oijk}{\sum }\left(z_{oi}^{k}n_{oi}^{k}t_{oi}^{k}+z_{ij}^{k}n_{ij}^{k}t_{ij}^{k}\right)\;$refers the complete transport time for the above two stages. The effect of punishment time $C_{4}=\underset{ik}{\sum }\theta_{i}^{k}\lambda_{i}^{k}\;$is the same as that of punishment cost, and the constraint model develops toward the goal of efficient allocation to meet emergency needs as far as possible.


(1) $$\begin{array}{l} MinimizeC=\sum_{i}^{}x_{i}f_{i}+\sum_{ik}^{}y_{i}^{k}s_{i}^{k}\;+\sum_{oijk}^{}\left(z_{oi}^{k}n_{oi}^{k}c_{oi}^{k}+z_{ij}^{k}n_{ij}^{k}c_{ij}^{k}\right)\;\\ \;+\sum_{ik}^{}\theta_{i}^{k}\lambda_{i}^{k}\; \end{array}$$

(2) $$\begin{array}{rcl} MinimizeT & = & \underset{i}{\sum }t_{i}+\underset{oijk}{\sum }\left(z_{oi}^{k}n_{oi}^{k}t_{oi}^{k}+z_{ij}^{k}n_{ij}^{k}t_{ij}^{k}\right)\;+\underset{ik}{\sum }\theta_{i}^{k}\lambda_{i}^{k}\; \end{array}$$

(3) $$\begin{array}{rc} Subject\;to & \underset{o}{\sum }z_{oi}^{k}n_{oi}^{k}=x_{i}y_{i}^{k}=\underset{j}{\sum }z_{ij}^{k}n_{ij}^{k},\forall i\in I,k\in K, \end{array}$$

(4) $$\begin{array}{r} d_{j}^{k}=\underset{j}{\sum }x_{i}z_{ij}^{k}n_{ij}^{k},\forall j\in J,k\in K, \end{array}$$

(5) $$\begin{array}{r} \underset{i}{\sum }x_{i}z_{oi}^{k}n_{oi}^{k}\leq h_{o}^{maxk},\forall i\in I,k\in K, \end{array}$$

(6) $$\begin{array}{r} n_{oi}^{k}\leq x_{i},n_{ij}^{k}\leq x_{i},\forall i\in I,k\in K, \end{array}$$

(7) $$\begin{array}{r} \underset{j}{\sum }d_{j}^{k}\leq h_{o}^{maxk},\forall k\in K, \end{array}$$

(8) $$\begin{array}{r} z_{oi}^{k},z_{ij}^{k}\in \left\{0,1\right\},\forall o\in O,i\in I,j\in J,k\in K, \end{array}$$

(9) $$\begin{array}{r} x_{i}\in \left\{0,1\right\},\forall i\in I, \end{array}$$

(10) $$\begin{array}{r} z_{oi}^{k},z_{ij}^{k},x_{i}\geq 0,\forall o\in O,i\in I,j\in J,k\in K, \end{array}$$

Note that (1) and (2) are objective functions, and (3)–(10) are constraints. The objective function (1) minimizes the allocation cost of emergency supplies *C* that includes the opening cost of mobile facilities service points, the storage cost of mobile facilities service points and transportation cost of mobile emergency supplies in the complete process of mobile emergency response. The objective function (2) minimizes the deployment time *T* of emergency supplies that includes the transport time of mobile emergency supplies and the preparation time of mobile emergency facilities. The constraint (3) indicates that the inflow quantity $\underset{o}{\sum }z_{oi}^{k}n_{oi}^{k}$ of a certain kind of materials at the service point of the selected facility should be conserved with the storage of $x_{i}y_{i}^{k}$. The outflow quantity $\underset{j}{\sum }z_{ij}^{k}n_{ij}^{k}$ of such materials at the service point is the loss occurred in the material turnover process that can be ignored. The intermediate service point has no right to detain emergency materials for self-deployment. The constraint (4) represents that demand $d_{j}^{k}$ of the emergency supplies demand point should be met by the multiple sources. The constraint (5) indicates that the number of materials $x_{i}z_{oi}^{k}n_{oi}^{k}$ issued by the mobile emergency supplies storage center should not be exceed the upper limit $h_{o}^{maxk}$ of its pre-storage for the mobile emergency supplies allocation system. The constraint (6) indicates that the selection of service points of mobile emergency facilities should be preset in advance for transferring the materials. The service points of mobile emergency facilities of $n_{oi}^{k}=1$ are eligible to participate in the transfer of mobile emergency supplies. Based on the conservation of materials in constraint (3), the constraint (7) indicates that the sum of the maximum demand $\underset{j}{\sum }d_{j}^{k}$ to meet any demand point should be less than the upper limit $h_{o}^{maxk}$ at the mobile emergency supplies storage center. This is required to prevent the system model failure caused by the mismatching of the quantity of materials at the upper and lower levels.

## Solution Algorithms

### Multi-Objective Transformation

The proposed model presented in the previous section attempts to minimize the cost and time that is challenging to coordinate directly. However, this should be integrated by considering the differentiated requirements of cost and time in the actual deployment of mobile emergency supplies. Due to different measurement units, the multi-objective optimization problem cannot be transformed into a single objective optimization problem by simple linear weight values. Although the percentage dimensionless method has been used to eliminate the units of measurement of sub-targets. Further, it is difficult to eliminate the order of magnitude differences between different targets. Therefore, this paper adopts another seed target unit elimination method. Let *C*^*R*^ = *C*/*C*^*m*^, and *T*^*R*^ = *T*/*T*^*m*^ be two multi-objective functions of *C*^*min*^and *T*^*min*^, respectively in a certain situation. The mobile emergency supplies mode minimizes the cost (regardless of the time factor) and time (regardless of the cost factors) through the optimal solutions to single-objective optimization problem. This is used to eliminate the two target measurement units. An order of magnitude difference can automatically adjust based on the data change. Based on the assumptions, decision-makers assign the importance degree to the two objectives in different situations. Let α be the importance degree value assigned to the cost objective function based on the actual situation, which is also known as the cost preference degree, then the objective function of the multi-objective mobile emergency supplies allocation model can be transformed into the following form:


(11) $$\begin{array}{r} minimize\;\alpha C{\;}^{R}+\left(1-\alpha \right)T^{R}=\alpha \frac{C}{C^{min}}+\left(1-\alpha \right)\frac{T}{T^{min}} \end{array}$$

### Shuffled Frog Leaping Algorithm Based on Material Allocation

Science the number of routes and vehicles can easily amplify the final possible outcome, the Shuffled Frog Leaping Algorithm (SFLA) is chosen as the solution scheme for obtaining the global solution of the proposed model. The SFLA has the advantages of simple concepts, fast computing speed and strong search coverage, which is helpful to avoid falling into local optimization. The scientific nature of the algorithm itself and the good matching degree with the model can also improve the global advantage of the search results. The hybrid leapfrog algorithm uses the frog group hunting mode for transmitting the information in the whole process. The algorithm takes advantages of the Genetic Algorithm (GA) and Particle Swarm Optimization (PSO) algorithm by combining global and local search information exchange. Thus, the local frog individuals are gradually optimized, and sorted through repeated ethnic groups of mold for information exchange from the local to global. This process continues until the preset convergence condition is satisfied. The specific algorithm steps are as follows:

Step 1: The determination model consists of three layers of stations, namely, the emergency supplies storage center *O* of the upper station, the emergency supplies service point *I* of the middle station, and the emergency supplies demand point *J* of the bottom station. Due to no restrictions imposed on any station number, the station name is not required to express as the genetic encoding. We consider those stations that allocate to determine the length of the gene encoding. In digital coding, each gene is represented through the character.
Step 2: Initialize the algorithm. Under the premise of considering the actual situation of material allocation, the size of the initial frog group was set as *N*, the number of groups is *a*, each group contains *b* frogs and satisfies *N* = *ab*, the maximum leapfrog stride allowed by the frog is *D*_*max*_, the number of local searches is *L*_*max*_, the global maximum number of iterations is *T*_*max*_. Further, *N* frogs were randomly generated to represent feasible emergency material allocation schemes. The *N* frogs are arranged in descending order from large to small according to the time and cost multi-objective planning adaptive values to form the initial population *N* = {*x*_1_, *x*_2_, *x*_3_, ⋯*x*_*n*_, }, where *x*_*i*_ refers to the *i*^*th*^ frog in the population, namely, the *i*^*th*^ type deployment scheme. Note that *X*_*G*_ refers to the optimal frog in the population, namely, the current optimal deployment scheme.
Step 3: Carry out local optimization and global optimization. The frog is divided into multiple child groups. In the process of grouping, the Number 1 frog points in group 1, number 2 frog points into group 2. The Numbers for *a* frog points into the group *a*, and number *a*+1 frog points to group 1, and so on, until the first *N* assigned to the end of the first group. Then, a local search was conducted within each group to determine the optimal frog (the best emergency material allocation plan) $x_{B}^{a}$ and the worst frog (the worst emergency material allocation plan) $x_{W}^{a}$ within the subgroup. The frog with the worst adaptation value for material allocation in the group is locked. Based on the preset position update leapfrog step length, and the difference between traditional SFLA, supplies allocation hybrid leapfrog algorithm needs to consider the actual material mixing in the process of rationality and beyond the model constraints. The deployment against supplies basic rules should be timely updated solution filter to delete, and meet the requirements of the new value, Then, the adaptive value of material allocation is recalculated as the independent variable of multi-objective function, and if the updated scheme is better, it replaces the worst solution in the group. If the above process has no improvement effect, *x*_*G*_ is again used to replace $x_{B}^{a}$ to perform the local search. If there is still no improvement, a new solution satisficing the requirements is directly generated to replace the worst frog $x_{W}^{a}$. After repeated local optimization of *L*_*max*_, the updated groups are remixed and arranged in descending order, and the optimized new emergency material allocation scheme in each sub-population is introduced into the whole world. The procedure started in the second step is repeated until the global maximum number of iterations *T*_*max*_ is met, and the optimal frog is obtained as output.

We compared our proposed algorithm with the traditional SFLA. The improvement of the Shuffled Frog Leaping Algorithm based on material allocation is mainly reflected in the improvement of the update strategy for material *x*_*W*_ allocation characteristics after local search. The frog jump update method is shown in (12), where *D* is a random number generated between 0 and 1, and the worst frog *x*_*W*_ performs frog jump randomly within the allowable range of maximum stride length *D*_*max*_. The best fitness solution *x*_*B*_ and the worst fitness solution *x*_*W*_ in the sub-population are needed to jointly update the worst frog, to seek the possibility of obtaining better fitness results. Subsequent updates to the characteristics of material allocation need to be carried out under the condition of following the basic logic of material allocation, and the worst fitness solution is more rapid in the process of approaching the best fitness solution. Instead of unified adjustment, the codes of each dimension of the worst emergency plan can be adjusted according to the positive and negative relationship of the codes corresponding to the best emergency plan, and the accuracy of local optimization can be improved.


(12) $$D=\{\begin{array}{c} min\left\{int\left[rand\left(x_{B}-x_{W}\right)\right],D_{max}\right\},x_{B}-x_{W}\geq 0,\\ \begin{array}{c} ,\\ max\{int\left[rand\left(x_{B}-x_{W}\right)\right],\\ \;{-D_{max}\}}^{x_{B}-x_{W}<0,x_{W}^{*}=x_{W}+D} \end{array} \end{array}$$

## Analysis of Examples

### Data Description

**Figure 1:**
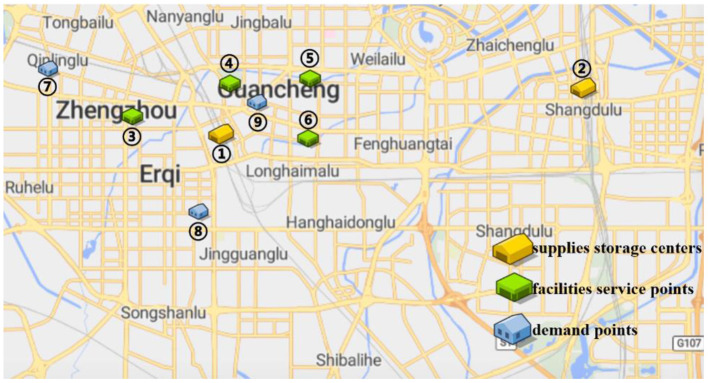
Locations of three types of mobile emergency stations in Zhengzhou.

In order to verify the adaptability of the multi-objective mobile emergency supplies allocation model to complex external conditions and its support to the location of service points of emergency facilities and emergency supplies scheduling, we consider the emergency rescue of rare sudden heavy rainfall in Zhengzhou, Henan Province, China in July 2021 as an example for analyses. The rainstorm lasted for at least 3 days and affected a wide area. According to the monitoring data of the China National Meteorological Observatory, the daily rainfall in central and northern Henan provinces, including Zhengzhou, Xinxiang, and Hebi, has exceeded the historical observation peak and affected 3,004,000 people in 103 counties. The precipitation in 1 h and 1 day in Zhengzhou exceeded the 60-year historical record since 1951. The waterlogging and mountain flood geological disaster has caused a great threat to people's life and property safety. Many citizens in Zhengzhou were badly affected and in urgent need of emergency supplies. In the optimization of deployment of multi-objective mobile emergency supplies, the alternative location of mobile emergency supplies storage center should meet the requirements of open terrain, convenient transportation, and abundant space. The coincidence degree of service site selection of mobile emergency facilities and the urban center should be considered to ensure the synchronous improvement of the overall coverage area and service capacity of each point. Therefore, 2 mobile emergency supplies storage centers at Zhengzhou Station and Zhengzhou Dong Station, 4 mobile emergency facilities service points at Bishagang Park, Zhengzhou People's Park, Zijing Mountain Park, and Shangdu Ruins Park, and 3 emergency supplies demand points Zhongyuan District, Erqi District and Guancheng District were set. We identify each station through the numbers 1,2,…9, respectively, their respective geographical location is shown in Figure 1, Table 1. The professional relief materials, life support materials, special supporting materials are numbered as *K*_1_, *K*_2_, *K*_3_. The transport time and unit transport cost among stations are shown in Table 2. Of course, A is the starting point number of transportation, B is the terminal number of transportation, *t*^*k*^ is the transportation time, and *c*^*k*^ is the unit transportation cost. The service point information of mobile emergency facilities is shown in Table 3. Note that notation *f*_*i*_ is the construction cost, *t*_*i*_ is the emergency turnaround time, and *s*_*i*_ is the unit storage cost. The information related to emergency supplies is shown in Table 4. Of course, $h_{o}^{max}$ is the maximum supply of storage center and $d_{i}^{max}$ is the demand of material demand point. In addition, the unit penalty coefficient is $\lambda_{j}^{k}=50$ for unmet demand of emergency supplies demands point.

**Table 1:** Geographical information of three types of mobile emergency stations in Zhengzhou.

**No**	**Name**	**Longitude**	**Latitude**	**No**	**Name**	**Longitude**	**Latitude**
1	Zhengzhou Station	113.654	34.747	6	Shangdu Ruins Park	113.685	34.747
2	Zhengzhou East Station	113.772	34.760	7	Zhongyuan District	113.597	34.765
3	Bishagang Park	113.624	34.752	8	Erqi District	113.646	34.723
4	Zhengzhou People's Park	113.656	34.761	9	Guancheng District	113.665	34.756
5	Zijing Mountain Park	113.682	34.763				

**Table 2:** Information of transportation time and unit transportation cost between stations.

**A**	**B**	**t^k^/min**	**c^k^/CNY**	**A**	**B**	**t^k^/min**	**c^k^/CNY**
1	3	13	3.0	3	9	13	3.5
1	4	14	2.5	4	7	15	4.5
1	5	23	4.0	4	8	18	4.5
1	6	21	3.5	4	9	6	2.0
2	3	28	9.8	5	7	18	4.6
2	4	33	8.2	5	8	24	4.6
2	5	35	5.4	5	9	11	2.0
2	6	29	7.0	6	7	22	4.6
3	7	9	2.5	6	8	21	3.8
3	8	16	4.0	6	9	6	1.5

**Table 3:** Mobile emergency facility service point information.

**Service point**	***f*_*i*_/CNY**	***t*_*i*_/min**	**Unit storage cost /CNY**		
			***K*_1_**	***K*_2_**	***K*_3_**
3	3,000	60	3.5	2	3
4	3,000	60	3.5	2	3
5	2,000	40	3.5	2	3
6	2,000	40	3.5	2	3

**Table 4:** Emergency supplies information.

		**K_1_**	**K_2_**	**K_3_**
$h_{o}^{max}$	1	500	650	600
	2	500	400	400
$d_{i}^{max}$	7	400	350	400
	8	300	35	300
	9	200	200	200

### Results Analysis

**Figure 2:**
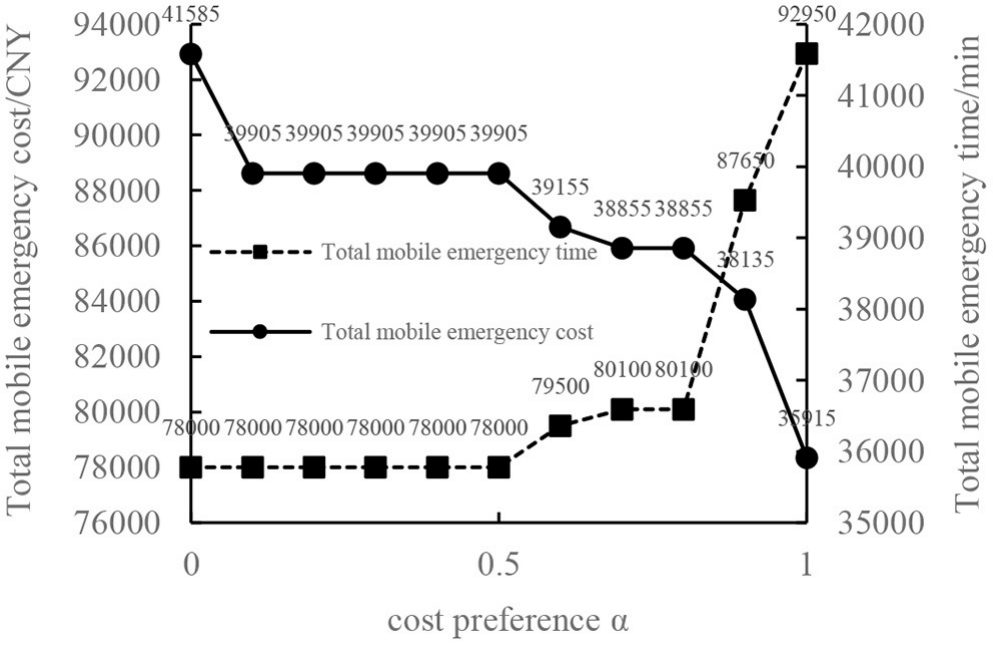
Mobile emergence cost and time variation under different cost preference degrees.

In order to verify the effectiveness of the multi-objective function, we consider the general applicability of the model in different situations. The cost preference is evaluated from 0 to 1 in ascending order of step size 0.1, and the Shuffled Frog Leaping Algorithm based on material allocation is used to optimize the solution step by step. The total number of frog population *N* = 100, the number of frog population *a* = 4 and the number of frog subpopulation *b* = 25 are set. The local search times *L*_*max*_ is set as 20, the overall maximum iteration times *T*_*max*_ is set as 50, and the maximum frog jump step length *D*_*max*_ is set as 30. The optimal allocation scheme of emergency supplies satisfying all material requirements is shown in Table 5, Figure 2. In the initial stage of a sudden disaster, the cost preference is low, so the decision-makers of mobile emergency response need to give priority to the urgency of emergency time and use two service points of emergency facilities (3, 6) in a concentrated and frequent way to ensure the safety of the lives and property of the affected people. When the cost preference degree rises to 0.9, the number of service points of mobile emergency facilities increases to three (3,5,6 and 4,5,6) indicating post-disaster emergency cost factors. This indicates that post-disaster emergency cost has been gradually attached importance to, the number of mobile emergency facilities has increased, and their distribution has also gradually expanded. It is worth noting that the total value of multi-objective programming reaches the maximum value when the cost preference degree is 0.5–0.6, which means that the maximum value of the multi-objective programming function can be guaranteed by giving equal attention to the cost factor and the time factor. Service point 3 has always been the service point with the largest flow indicating that there are core stations in the mobilization process of mobile emergency supplies. They are not easily changed by external factors. Exploring the core stations is necessary to work in the planning of mobile emergency supplies transportation.

**Table 5:** Optimal emergency plan under different cost preference degrees.

**α**	**Participating service points**	**The most used service point**	**Multi-objective result**
0	3,6	3	1
0.1	3,6	3	1.003426
0.2	3,6	3	1.006831
0.3	3,6	3	1.010278
0.	3,6	3	1.013705
0.5	3,6	3	1.017131
0.6	3,6	3	1.020194
0.7	3,6	3	1.019258
0.8	3,6	3	1.018242
0.9	3,5,6	3	1.015524
1	3,4,5	3	1

When the cost preference gradually increases, the optimal value of mobile emergency cost gradually decreases, but the optimal value of mobile emergency time keeps increasing, as shown in Figure 1, which is basically consistent with the actual situation of mobile emergency material allocation. Theoretical research can be combined with the actual mobile emergency material allocation according to the given concluding remarks. Different values of cost preference degrees are used to map different stages in the real mobile emergency process. From Table 6, corresponding to α = 0.2 the mobile emergency response time is given priority in the initial stage of mobile emergency response. The shortest emergency response time is regarded as the only constraint for the allocation and transportation of mobile emergency supplies. The mobile emergency transport network is rapidly activated to carry out the transportation of various emergency supplies, and striving to control the impact of disasters at all cost. In the middle phase of mobile emergency response, corresponding to α = 0.6 shown in Table 6, the cost and time of mobile emergency response have reached a relative balance. The professional relief materials have been transported in place, and life support materials need to supply steadily. The urgent requirements of mobile emergency response materials transportation and the cost constraints should be considered from the actual point of view. In the late phase of mobile emergency response, corresponding to α = 0.9 in Table 6, the cost preference is in ascending order. The mobile emergency supplies deployment comes into the finishing touches. The professional and life safeguard aid have been basically saturated. These create a complete set of special class supplies that are needed for bulk supply emergency recovery. This stage usually takes long-term planning that how mobile emergency funds are to be used.

**Table 6:** Optimal allocation routes and quantities of mobile emergency materials under three cost preference.

	**α = 0.2**	**α = 0.6**	**α = 0.9**
	***C* = 39905, *T* = 78000**	***C* = 39115, *T* = 79500**	***C* = 38135, *T* = 87650**
*K*_1_	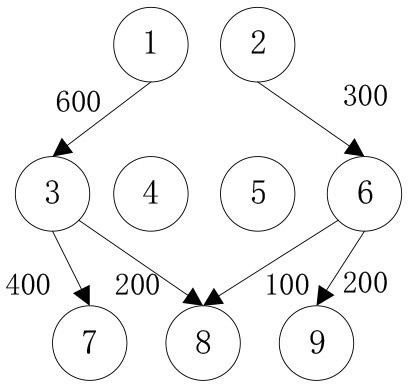	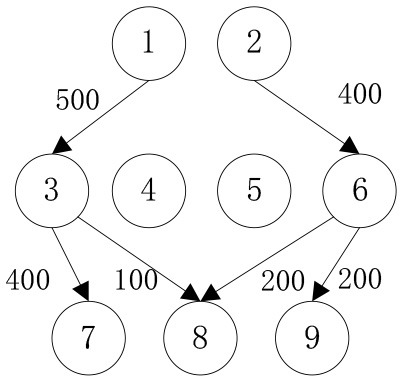	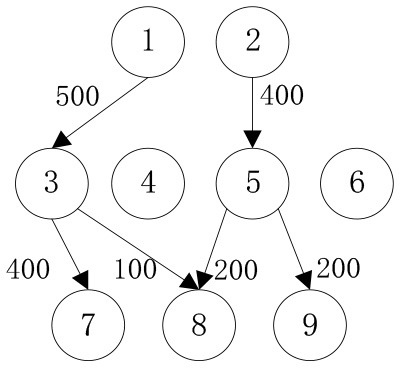
*K*_2_	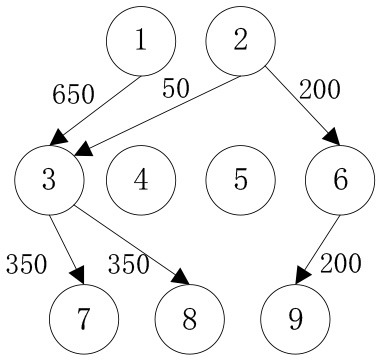	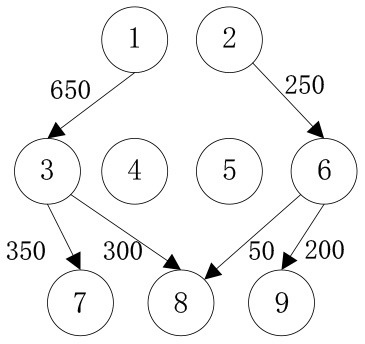	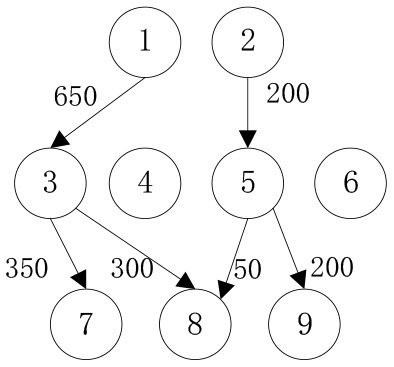
*K*_3_	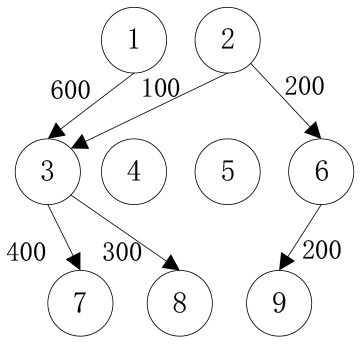	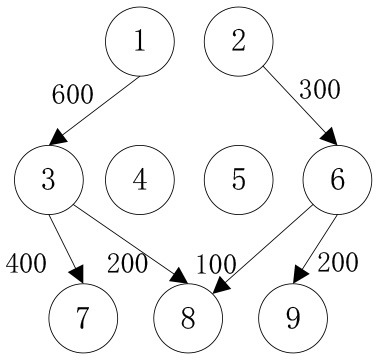	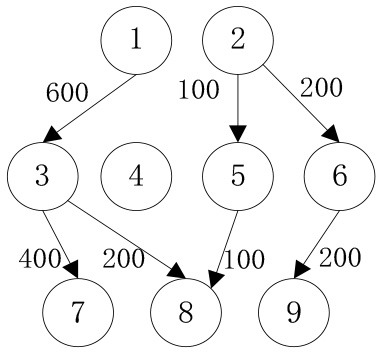

In addition, combined with mobile emergency facilities service point of geographic information, we can analyze the mobile emergency subject location that affects mobile emergency supplies allocation at all levels based on the three cases. In the optimal deployment of the path, we find that the change of the cost and time preference cannot shake 1 (Zhengzhou Station) and 3 (Bishagang Park) as the core of the mobile emergency supplies allocate participants. In sharp contrast, 2 (Zhengzhou People's Park) has not been involved in the material allocation process. By analyzing the external conditions, we can see that the more coincident the geographic location with the center of a mobile emergency service area, the easier the emergency stations are to be included in the optimal allocation path. At the same time, when the geographic location of two stations with close objective configurations is similar (2,3 locations are 6 km apart), the weaker stations are very easy to be ignored and abandoned completely in an early emergency. Therefore, under the precondition of fixed location of mobile emergency materials reserve point, mobile emergency setup service point should give priority to setting up the whole regional center of mobile emergency services, and avoid repetitive set up in a small range to ensure the effect of mobile emergency materials allocation.

## Conclusion

We compared our proposed model with the traditional emergency material allocation mode. The mobile emergency facilities provide a way to improve the decision-making efficiency of emergency material allocation and reduce the cost of material allocation by their flexibility and autonomy. To get close to the actual situation of emergency rescue and meet the actual needs of emergency rescue, this paper constructs the multi-objective mobile emergency material allocation model based on the exploration of the working mechanism of mobile emergency facilities. We consider the factors such as unified data order and apply the method of transforming multi-objective planning into single objective optimal value for improving the traditional SFLA. We design a Shuffled Frog Leaping Algorithm based on material allocation, and strengthen the screening process after local optimization. We ensure that the logic of the final mobile emergency material allocation scheme is feasible. Finally, we take the “21.7” Torrential Rain and flood disaster in Henan Province as an example to verify the effectiveness of the model and algorithm. Further, we analyze and discuss the impact of cost preference, mobile emergency facility location and mobile material allocation on the overall mobile emergency material allocation and transportation, and provide the corresponding values of cost preference in different stages of mobile emergency and suggestions for mobile emergency facility location and emergency material allocation and transportation. For future research, we can consider robust optimization for unknown parameters with uncertainty such as emergency material demand and emergency material transportation time, to better fit the actual situation of mobile emergency, which is the feasible direction of further research.

## Data Availability Statement

The datasets presented in this study can be found in online repositories. The names of the repository/repositories and accession number(s) can be found in the article/supplementary material.

## Author Contributions

All authors contributed to the conception and writing of the paper and read and agreed to the published version of the manuscript.

## Funding

This research was funded by National Social Science Fund of China, Grant Number 21BGL200.

## Conflict of Interest

The authors declare that the research was conducted in the absence of any commercial or financial relationships that could be construed as a potential conflict of interest.

## Publisher's Note

All claims expressed in this article are solely those of the authors and do not necessarily represent those of their affiliated organizations, or those of the publisher, the editors and the reviewers. Any product that may be evaluated in this article, or claim that may be made by its manufacturer, is not guaranteed or endorsed by the publisher.
